# Study of Nutritional and Organoleptic Quality of Formulated Juices from Jujube (*Ziziphus lotus* L.) and Dates (*Phoenix dactylifera* L.) Fruits

**DOI:** 10.1155/2020/9872185

**Published:** 2020-03-31

**Authors:** Meryem Benidir, Soukaina El Massoudi, Lahsen El Ghadraoui, Abderrahim Lazraq, Meryem Benjelloun, Faouzi Errachidi

**Affiliations:** ^1^Department of Biology, Laboratory of Functional Ecology and Environment, Faculty of Science and Technology, Sidi Mohammed Ben Abdellah Fes University, P.O. Box 2202, Fes, Morocco; ^2^Laboratory of Pathophysiology and Nutrition and Environment, Faculty of Medicine and Pharmacy, Sidi Mohammed Ben Abdellah University, P.O. Box 1893, Fes, Morocco

## Abstract

The present work aims to elaborate many juice formulas (F1 to F8) from two dried fruits (jujubes: *Ziziphus lotus* L. and dates: *Phoenix dactylifera* L.). Physicochemical and biochemical characterization of the formula juices shows that juices rich in dates fruits (F1, F3, F5, and F7) are loaded, on average, in total sugars (129.5 g/l), proteins (3.02 g/l), lipids (1.08 g/l), and carotenoid (0.02 mg/l), while juices rich in jujube fruits (F2, F4, F6, and F8) are overloaded, on average, in phenolic compounds (697 mg/l), flavonoids (6.32 mg/l), condensed tannins (2.1 mg/l), hydrolysable tannins (359.5 mg/l), and viscosity (1.062 mm 10–3 s). All formulations developed have a pH that rotates between 5.12 and 5.20. Total antioxidant capacity (CAT) reveals that formulations F1, F3, F5, and F7 show a strong activity compared to the other formulas. The DPPH test shows that all formulated juices have the same antioxidant profile with IC_50_ values lower than the template (BHT and Vit C). The FRAP test reveals that F2, F4, F6, and F8 formulas have a strong reducing power. Organoleptic evaluation by a jury shows that F4 formula is the best in terms of odor, aroma, and aftertaste agreeability.

## 1. Introduction

Fruit juice consumption overall the world has increased in recent years, probably due to public perception of juices as a healthy natural source of nutrients and augmented public interest in health issues. The current trend is oriented likewise towards juice from dried fruit which constitutes a concentrated form of fresh fruits prepared by different drying techniques [1]. Dried fruits in smaller serving sizes, after stabilization by elimination of a large amount of water, are considered nutritionally equivalent to fresh fruits in current dietary recommendations in different countries [2–4].

Currently, scientific society highlights the tragic role of uncontrollable oxidative process induced by reactive oxygen species (ROS). These oxidants are the direct cause of various pathologies such as cancer, diabetes, Alzheimer's, and cardiovascular diseases [5, 6]. Whatever the case, risk is increased with their accumulation (ROS) in the body following a radical reaction chain which degrades vital biological molecules, namely, proteins, lipids, and DNA [7, 8]. Given increasing diseases caused by oxidative stress, natural antioxidants are the subject of much research and a new tendency towards secondary metabolites exploitation, generally phenolic compounds, used particularly in health and chronic diseases and in food industry. All this meant to fight against oxidative stress and its associated pathologies.

Many scientific studies point out that people who regularly consume large amounts of dried fruit or their derivatives have a lower rate of various types of cancer, cardiovascular disease, obesity, type 2 diabetes, and other chronic diseases [9]. Therefore, dried fruits and their derivatives should be consumed daily in order to take full advantage of the nutrients, health-promoting phytochemicals, and antioxidants they contain, as well as their unique and desirable taste and aroma. Due to the presence of antioxidant and anti-inflammatory substances, dried fruits can prevent carcinogenesis.

Fruit manufacturing induces physical and chemical modifications which reduce quality [10]. The ability of industry to provide the consumer with a nutritious and healthy fruit product is highly dependent on the nature of the characteristics changes that occur during processing. Accordingly, production and trade of dried fruits and their derivatives present major challenges, such as problems with technology processing, food safety aspects, and quality assurance. This work targets the introduction of innovative developments in the field of valuing dried fruits in Morocco and provides a new concept which may reduce the deterioration of these fruits in conventional processes. As a valuation model, we have targeted two dried fruits from the Moroccan terroir, namely, dates and jujube fruits.

The jujube (*Ziziphus lotus* L.), commonly called “Sedra” in Morocco, as a local product targeted by this study, grows on all the southern shores of the Mediterranean [11]. As traditional medicinal plants, it is recommended for digestive disorders, weakness, liver diseases, obesity, urinary disorders, diabetes, skin infections, fever, diarrhea, and insomnia [12–14]. Other Ziziphus fruits effects have been reported as antiaging, antitumoral, anti-inflammatory, antiulcerogenic, and antibacterial [15, 16]. Although they are multipurpose and have an undeniable ecological interest, these jujube trees have become rare in the north of Africa, even threatened with disappearance [17].

Dates are an important fruit, “An Emerging Medicinal Food,” especially in many African, Middle Eastern, and Asian countries. Besides its local and regional commercial value, date palm plays an important role in the diet and social life of communities across the oases. In recent years, this fruit has gained significant importance in global commerce as well. During the last two decades, the world production of dates has more than doubled.

Date processing industries are producing various date products like date paste, syrup, jam, vinegar, and so on [18–20]. However, date fruit has not been afforded its due importance to a scale similar to some other subtropical fruits. There is a vast potential for processing dates into value added products. Equally to our knowledge, very limited publications have been focused on jujube in food technology [21]. The last author and his collaborators have integrated this plant fruit in biscuit formulation. Our contribution is centered to another sector, namely, juices formulation and technology. In this perspective, this work focuses on a valuation of jujube combined with dates fruits in food industry through high nutritional value exotic juice formulation. Thus, this study proposes evaluation of juices formula rich in phenolic compounds and having a greater antioxidant activity. Medicinal plants, and their derivatives (essential oils, flavonoids, etc.), are a valuable source of antioxidant and antimicrobial agents [22]. Our perception targets the convergence of products naturally rich in antioxidants and preservatives (essential oils and certain medicinal plants having a good taste as cinnamon) to improve astringency which can be suggested by the mixture of two dry fruits (jujube and dates).

## 2. Material and Methods

### 2.1. Plant Material

Fruits used in this study are *Ziziphus lotus* L. (jujube) and *Phoenix dactylifera* L. (date: *tarzawa* variety). Both of them were purchased from dry fruits national market in Fes and also were harvested during the month of September 2018.

### 2.2. Methods

#### 2.2.1. Exotic Juice Preparation

To prepare juices formula, jujube fruits are pitted and grounded with an electric mixer and then sieved to obtain homogeneous fine powder. As for the dates once pitted, they are crushed to obtain a paw. The transformed fruits are mixed in order to develop formula according to experimental design method (8 formulas) by decoction of 1 kg of the mixture in 4 liters of distilled water during 20 min (80°C) and filtration by sterile gauze. After cooling the juices, anise essential oil and powdered cinnamon have been added to the different formulations (as shown in Table 1). Variables have a significant effect on organoleptic characteristics according to the following factors:

- Date/jujube ratio (40% (solid date)/60% (solid jujube) or 60% (solid date)/40% (solid jujube))
- Preservative concentration (anise essential oil) used to stabilize juices and inhibit microbiological alteration (10 *μ*l/100 ml or 30 *μ*l/100 ml)
- Flavoring agent concentration (cinnamon) (10 mg/100 ml or 30 mg/100 ml)

Prepared juices were evaluated by a jury (27 tasters) to determine their organoleptic criteria evaluation. The tasting test was developed according to [23].

## 3. Quantitative Analysis of Elaborated Juices

### 3.1. Biochemical Characterization

#### 3.1.1. Macronutrients Determination

In this part, we have determined total and reducing sugars, protein, and lipids content in elaborate formula. Total and reducing sugars were evaluated using dinitrosalicylic acid (DNS), on the formation of a chromatophore between the reagent and the endings reducing sugars according to [24]. Concentrations are deduced from a calibration curve developed from a stock solution of 1 g/L of glucose. Protein content was determined by colorimetric assay using Folin–Ciocalteu reagent [25]. Protein concentrations are determined using a standard curve constructed from Bovine Serum Albumin (BSA) stock solution of 0.1 g/L. Lipid content was determined by cold maceration according to [26].

#### 3.1.2. Micronutrients Determination

This part is centered on micronutrient determination; we target the qualification of vitamin C, carotenoids, phenolic compounds, flavonoids, and tannins (condensed and hydrolysable) in elaborated formula.

Vitamin C content was determined by colorimetric assay [27]. Vitamins B1 and 2 and minerals analyses were done using standard methods of Association of Official Analytical Chemists [28]. Carotenoid content was performed according to [29]. Concentrations are determined using a calibration curve prepared from *β*-carotenes stock solution of 0.1 mg/ml. Phenolic compounds contents was carried out using the Folin–Ciocalteu reagent, according to the method described by [30]. Concentrations are expressed in *μ*g equivalent of gallic acid per milliliter of the juice (*μ*g EG A/ml). Flavonoid content was performed according to the aluminum trichloride (AlCl3) method described by Barros with some modification [31]. Concentrations are expressed in *μ*g equivalent of quercetin per milliliter of the juice (*μ*g EQ/ml). Condensed tannins determination was carried out using the method developed by [32]. Hydrolysable tannins are determined by method of [33] using reactive of potassium iodate. Concentrations are expressed in *μ*g equivalent of tannic acid per milliliter of the juice (*μ*g TA/ml).

### 3.2. Physicochemical Analysis

pH measurements were carried out using a pH meter (Thermo Orion 3 Star). Juices viscosities were determined using a capillary viscometer (Ostwald) at 24°C according to the method described by [34].

## 4. Antioxidant Activity

In this study we have evaluated antioxidant activity of all juices deluded 100 times by three techniques to scan the antioxidant power of several compounds in elaborate juices.

Total antioxidant capacity (TAC) was done according to the method cited by [35] with some modification using ammonium phosphomolybdate reagent. Results obtained are expressed in mg equivalent of vitamin C per ml. Free radical DPPH was used to determine the antiradical capacity of juices (diluted 100 times) by a redox reaction according to the method described by [36]. Kinetics activities were used to determine concentrations which correspond to 50% inhibition (IC_50_). Antioxidants activity is expressed in BHT % and vitamin C % equivalent.

The third technique is used to measure the capacity of samples to reduce ferric iron (Fe^3+^) to potassium ferrocyanide (Fe^2+^) [37]. Ascorbic acid is used as a positive control in this dosage.

## 5. Results and Discussion

### 5.1. Quantitative Analysis

#### 5.1.1. Biochemical and Physicochemical Characterization

Before proceeding to the formulation of the juices, we carried out an analysis of the raw materials used in the formulation of the juice prepared by the jujube and the dates fruits. The results obtained are illustrated in Table 2.

The results obtained show a certain complementarity between the two fruits targeted by the study. Thus, we note that the fruits of the jujube (*Ziziphus lotus*) are rich in phenolic compounds formed essentially by tannins and flavonoids while date fruits (*Phoenix dactylifera* L.) are richer in sugars, vitamins (Vit C, Vit B1, and Vit B2), and trace elements. The chemical composition of the two fruits is favorable for the preparation of a combined juice. The percentages of raw materials combined according to the experiment plan developed (Table 1) were the subject of more targeted analysis to assess the antioxidant (carotenoids and vitamin C) and antimicrobial (flavonoids and tannins) potential.

Biochemical and physicochemical analysis of formulated juices are summarized in Table 3.

Obtained results (Table 3) show that total sugars mean concentration is higher (129.50 g/l ± 0.24) in formulas F1, F3, F5, and F7 which have a ratio D/J > 1, whereas formulas F2, F4, F6, and F8 (D/J < 1) have lowest mean concentration (99 g/l ± 0.01). This is explained by the richness of dates fruits in sugars compared to jujube. This justifies our advantageous nutritional complementation of date-jujube mixture. This concept was also targeted by an Algerian research group in the field of biscuits [21]. Their research revealed that total sugar content in jujube was 24.29%. In return, [38] in Algeria found lower concentrations (10.55%) in jujube pulp fruits, and this difference observed could be due to changes in climate conditions between north and south of Algeria. On the other hand, dates are richer in total sugars which vary between 70 and 90% [39, 40]. High content of soluble sugars in dates gives them an appreciable contribution in formulated juice, as well as the possibility of transforming them into industrial products such as biscuits, syrups, juices, jams, marmalades, and jellies. In addition, analysis shows that formulations F1, F3, F5, and F7 recorded high average levels of proteins and lipids with averages of 3.2 g/l ± 0.02 and 1.1 g/l ± 0.01, respectively. In contrast, formulations F2, F4, F6, and F8 showed low mean concentrations of 2.28 g/l ± 0.04 of proteins and 0.46 g/l ± 0.01 of lipids. Results of [21] are different from ours, which revealed that jujube contained relatively small amounts of lipids (0.84%) and protein (1.43%); this difference could be due to changes in climate conditions between Algeria and Morocco. So, our results were almost consistent with value (2.10%) found by [41] in jujube pulp. Date fruit contains a small amount of lipids which vary between 0.43 and 3% [42–44]. Concerning micronutrients content, results obtained show that all formulations have almost the same average concentrations of carotenoids (0.02 mg/l ± 0.005). Having a report D/J < 1, high average contents of flavonoids, condensed tannins, and hydrolysable tannins are with values of 6.33 mg/l ± 0.02 EQ, 2.1 mg/l ± 0.01, and 359.5 mg/l ± 0.02 EAT, respectively. On the other hand, F2, F4, F6, and F8 formulas showed high average concentrations of phenolic compounds (64.57 mg/l ± 0.03 EAG). A study done by [45] revealed that the aqueous extract of jujube fruit is very rich in phenolic compounds (285.19 mg EAG/100 g dry extract). So, contribution of jujube formulations is, therefore, an enrichment in phenolic compounds. Moreover, physicochemical parameters demonstrate that all prepared formulations have a pH value which varies from 5.11 ± 0.02 to 5.19 ± 0.02, whereas viscosity average of formulations F2, F4, F6, and F8 is very high: 1.062 ± 0.03 mm 10^−3^/s. Compared to the other formulations, F1, F3, F5, and F7 have low values (0.247 ± 0.01 mm 10^−3^/s). From these results, we can conclude that fruit jujube increases viscosity of the juice because it is rich in pectin substance at concentration between 0.57 and 2.79% [46].

Biochemical and physicochemical characterization indicates that the complementation concept (jujube and dates) targets valuing existing excess sugars in dates and phenolic compounds of jujubes, in order to obtain a juice of high nutritive, therapeutic, and organoleptic values. A research group [47] carried out a physicochemical and sensory characterization of various formulations of apple and pineapple juice. This study shows that phenolic compounds concentration in prepared juices is lower (0.6 and 1.8 mg/ml) when compared to our elaborate juices. Vit C content is between 8.65 and 9.9 mg/l. Protein content was observed between 2.23 and 3.16 g/l, as well as lipids which showed lower concentrations values when compared to our prepared juices. Therefore, analysis carried out showed that all the juices tested have a pH that rotates between 5.11 and 5.19.

### 5.2. Formulated Juices Antioxidant Activity

To verify the results obtained in Table 3, we analyzed by three different methods the antioxidant activity in order to evaluate the spectrum of secondary metabolites (phenolic compounds, flavonoids, tannins, etc.) of the formulated juices.

#### 5.2.1. Total Antioxidant Capacity (TAC)

**Figure 1:**
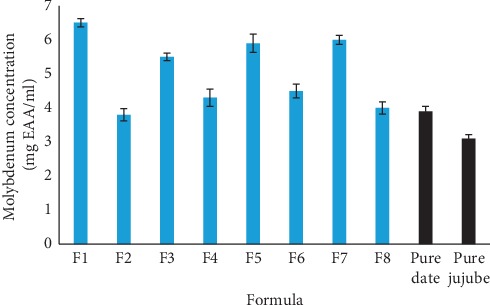
Total antioxidant capacities of formulated juices after dilution (100x).

Formulated juices total antioxidant capacities expressed in equivalent ascorbic acid (vitamin C) are represented in Figure 1. Juices total antioxidant capacity showed variability according to the formula elaborated. We noticed that diluted formulas (F1, F3, F5, and F7), in the case where ratio of dates/jujube > 1, showed a strong TAC, when compared to F2, F4, F6, and F8 juices (having report dates/jujube < 1). Antioxidant capacity observed in tested juices could be explained by their richness in phenolic compounds (flavonoids and tannins). Several studies have shown that samples antioxidant capacity is related to other molecules rather than phenolic compounds such as ascorbic acid or carotenoids, which means that strong antioxidant capacity recorded in the formula having ratio of dates/jujube > 1, which are richer in carotenoids (Table 3). References [48, 49] have also shown that antioxidant activity can be affected by many factors including phenolic compounds structure and synergistic interactions with various antioxidants. According to results obtained, ingredients introduced (essential oil and cinnamon) in formula do not have any antioxidant activity (TAC). So, observed antioxidant activity is due to bioactive molecules of two dry fruits (carotenoids, flavonoids, tannins condensed, and tannins hydrolysable). Then, dates contribute qualitatively of bioactive molecules to antioxidant activity while jujubes act quantitatively. The templates of this experiment are jujube fruit and dates juices that have lower values than those of formulated juices.

#### 5.2.2. Antiradical Activity by the 2,2-Diphenyl-1-picrylhydrazyl (DPPH) Method

Juices antioxidant activities done by DPPH method are shown in Table 4. Inhibition percentages are reflected by the complete discoloration of violet-yellow radical. Therefore, we found that free radical inhibition (DPPH) percentage increases with concentrations of different juices diluted and templates (BHT and Vit C) tested. So, these formulas possess an antiradical dose-dependent activity. Thus, we have detected that the percentage of free radical inhibition for juices is higher than that of the template (BHT and Vit C) for all the concentrations studied. The strongest antioxidant activity noted in all the formulas developed could be due to the synergistic effect of bioactive molecules [50, 51] existing in jujube and dates fruits. According to several studies, antiradical capacity is proportional to the contents of the polyphenols portrayed in the samples tested. In addition, rate of antiradical power of the varieties studied does not correspond to their total content of total phenols but rather correspond to the flavonoid levels [38, 52, 53]. It should be noted that this test is more suitable for polar molecules because the reaction medium is polar (the DPPH was prepared by methanol).

Antioxidant capacity of different juices was determined from the IC_50_, which is the concentration needed to reduce 50% maximal DPPH radical. The lower IC_50_ value corresponds to high antioxidant activity [54]. We determined IC_50_ from regression equations of the graphs (Table 4). According to the obtained results, recorded IC_50_ values of templates (BHT and Vit C) are 0.09 and 0.06 mg/ml, respectively. Their values are lower than those of formulated juices which presented IC_50_ between 0.015 and 0.017 mg/ml. According to results obtained, DPPH method essential oil has a little effect on inhibition of this free radical, because we noticed that, for the maximum concentrations of essential oil, the value of IC_50_ was 3 mg/ml. Pure noncombined juices present higher IC_50_ values when compared to formulated juices, having values of 0.02 and 0.03, respectively, for date and jujube juices.

#### 5.2.3. Antioxidant Activity of Juices by the Method of FRAP

**Figure 2:**
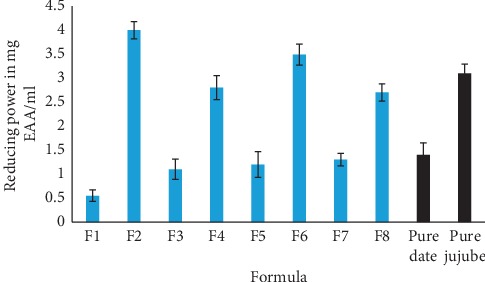
Antioxidant activity of juices formulated and diluted (100x).

In order to inquire about antioxidant activity of formulated juices, we used a 3^rd^ method (FRAP). This latter is commonly used to study antioxidant capacity in plants to evaluate the antioxidant activity of nonpolar molecules. The reducing power of elaborate juices is determined by the ability of their antioxidant molecules to reduce ferric (Fe^3+^) ions to ferrous (Fe^2+^) using the FRAP reagent. Antioxidant activities done by the FRAP method are shown in Figure 2. From these results, we found that all formulas developed have the same antioxidant profile, while formulas F2, F4, F6, and F8 show a higher reducing power compared to other juices (F1, F3, F5, and F7).

From antioxidant activity results of the juices by the three techniques, we find that all the formulas elaborated showed approximately the same antioxidant profile. This activity could be due mainly to the richness of juices in phenolic compounds and other bioactive molecules. It is well established that antioxidant activity is positively correlated with the concentration and structure of the polyphenols. Generally, polyphenols with a high number of hydroxyl groups have highest antioxidant activity [51], due to their ability to give more atoms to stabilize free radicals [52]. Based on this, we can conclude that the more juice is rich in total polyphenols the more marked its antioxidant power is. These results confirm those obtained by DPPH method. Figure 2 confirms that the jujube fruit contributes massively to the increase in antioxidant activities of formulated juices (F2, F4, F6, and F8).

## 6. Sensory Analysis of Formulated Juices

The results of the sensory analysis are shown in Table 5.

Results obtained show that the characters evaluated in this test can be subdivided into visual, olfactory, and taste criteria. For visual criteria (color, clarity, viscosity, texture, and gloss), characteristics of F8 revealed the most important values (7.60, 5.97, and 6.40, respectively). On the other hand, for texture, F7 formulation is the most representative by a value equal to 6.60, and finally for gloss the maximum value of 20.95 was observed in F4. Concerning the olfactory criteria (odor intensity and smell agreeableness), F7 formulation shows a high odor intensity (6.60) when compared to the other evaluated formula and for smell agreeableness F4 formulation was more pleasant at the odor level (8.52). Concerning the taste criteria (aroma agreeability, sugar/acid Equilibrium, bitterness, astringency, sugar intensity, and aftertaste) for the six criteria, F4 shows the most with, respectively, 9.64, 10.78, 8.24, 9.30, 9.49, and 9.45. From these results, we can conclude that F4 formulation is the best organoleptic value. A group of researchers [54] formulated several date-based milk drinks and orange concentrate with high dates. According to the tasting test carried out, we have retained one of the drinks for its organoleptic and sensory qualities (sugar/acid equilibrium, average sugar intensity, and a pleasant aftertaste).

**Figure 3:**
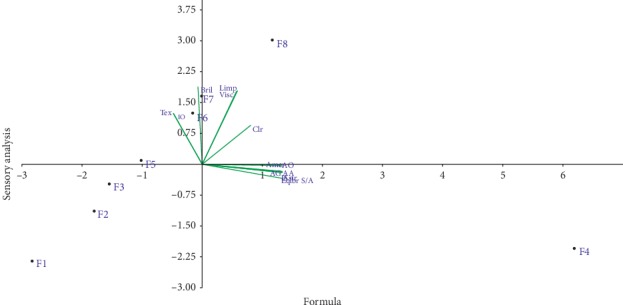
Principal composition analysis (PCA) of sensory analysis of formulated juices. Clr: color; Limp: limpidity; Visc: viscosity; Tex: texture; Bril: gloss; AO: smell agreeableness; AA: aroma agreeability; IS: sugar intensity; AG: aftertaste; Amer: bitterness; Astr: astringency; Eqlbr S/A: equilibrium of sugar/acid.

Dimensional analysis in Figure 3 of the data from Table 5 shows that the formulation F4 contributes positively to the component 1 and negatively to the component 2 and it correlates more with smell agreeableness (AO), aftertaste (AG), aroma agreeability (AA), and sugar/acid equilibrium (Eqlbr S/A). F7 and F8 contribute positively to components 1 and 2; in addition they are more correlated with color, viscosity, gloss, and limpidity. F1, F2, and F3 contribute negatively to components 1 and 2; also they are no longer correlated with any organoleptic criteria. F5 contributes negatively to component 1 and positively to component 2; furthermore it is correlated more with texture and sugar intensity. From the analysis of the results and from the pleasantness point of view of flavor and aftertaste, the best formulation is F4.

## 7. Conclusion

Primary objective of this study targeted biochemical and physicochemical analysis of juices based on *Ziziphus lotus* L. and *Phoenix dactylifera* L. Formulated juices analysis allowed us to confirm that *Ziziphus lotus* L. rich juices are characterized by high levels of phenolic compounds, flavonoids, condensed tannins, and a high viscosity. Juices rich in *Phoenix dactylifera* L. are characterized by high concentrations of total sugars, lipids, and proteins; the complementarity between the two dry fruits studied seems remarkable and could be used in food industry to develop other food products of high nutritional value. Elaborated juices antioxidant activities permitted us to obtain interesting results. Indeed, the three tests (CAT, DPPH, and FRAP) reveal that almost all formulas have a potent antiradical activity, so the elaborated juices can be consumed to fight against certain chronic diseases due to free radicals (ROS) (cardiovascular diseases, etc.). Sensory analysis juices formulas elaborated from *Ziziphus lotus* L. and *Phoenix dactylifera* L. allowed us to obtain interesting results and they could be exploited in food industry. All of these primary results obtained targeted valorization of these two dry fruits (jujube and dates). Therefore, it is important to study microbiological stability of the juices formulated during storage and thus the effect of pasteurization on the organoleptic characteristics of juices. Others pharmacological studies (pharmaceutical and pharmacodynamics) are planned to reveal the antioxidant activity of intracellular developed juices.

## Figures and Tables

**Table 1:** Formulated juices preparation.

Formula	J/D mixture ratio	EO (*μ*l)	Cinnamon (mg)
F1	40/60	10	10
F2	60/40	10	10
F3	40/60	30	10
F4	60/40	30	10
F5	40/60	10	30
F6	60/40	10	30
F7	40/60	30	30
F8	60/40	30	30

D: date; J: jujube; EO: essential oil.

**Table 2:** Distribution and contents of major bioactive, vitamins, and minerals compounds in jujube and date pulp.

		Analysis/fruits	Date pulp^*∗*^	Jujube pulp
g/100 g		Moisture	19	8.93
		Sugars	72.2	32.34
		Proteins	2.6	1.7
		Lipid	0.5	0.3
		Phenolic compounds	0.36	5.53
		Ash	1.7	1.57

mg/100 g		Vit B1	0.11	0.039
		Vit B2	0.12	0.08
		Vit C	6.31	0.6
		Ca	70.2	490
		Cu	0.7	nd
		Mg	64.5	11.2
		Mn	0.33	2.17
		Fer	1.72	2.1
		P	58.33	26.5
		K	536.97	118
		Na	32.1	3.6
		Zn	2.8	0.44

^*∗*^ *Tarzawa* variety.

**Table 3:** Biochemical (macronutrients and micronutrients) and physicochemical (PC) characterization of formulated juices of jujube and date fruits.

Analysis/formulas	F1	F2	F3	F4	F5	F6	F7	F8
Total sugars (g/l)	131	93	128	101	129	103	130	98
Reducing sugars (g/l)	98.76	69.75	99	75.75	94.83	77.25	95.65	73.5
Protein content (g/l)	3.12	2.42	3.23	2.04	2.97	2.24	3.35	2.42
Lipid (g/l)	1.20	0.46	0.97	0.57	1.10	0.37	1.05	0.42
Phenolic compounds (mg/l)	565	805	537	678	488	666	540	639
Vitamin C (mg/l)	9.36	8.66	10	8.9	9.8	8.56	10.4	8.5
Carotenoids (mg/l)	0.03	0.02	0.02	0.01	0.02	0.02	0.02	0.02
Flavonoids (mg/l)	2.70	7.60	4.00	5.90	5.90	5.10	4.60	6.70
Condensed tannins (mg/l)	0.93	2.37	1.39	1.921	1.75	1.903	1.97	2.22
Hydrolysable tannins (mg/l)	215	392	241	355	221	342	209	349
Viscosity (mm 10−3/s)	0.15	1.09	0.27	1.17	0.22	1.21	0.35	0.78
pH	5.43	4.94	4.98	5.03	5.13	5.35	5.23	5.15

**Table 4:** Antioxidant potency (expressed as IC_50_) of reference antioxidants and formulated juices.

Formula	IC_50_ (mg/ml)	J/D (g of pulp powder/100 ml of water) ratio; EO (*μ*l);
Cinnamon (mg)		
F1	0.017	40/60; 10; 10
F2	0.017	60/40; 10; 10
F3	0.01688	40/60; 30; 10
F4	0.01688	60/40; 30; 10
F5	0.017	40/60; 10; 30
F6	0.016	60/40; 10; 30
F7	0.01688	40/60; 30; 30
F8	0.015	60/40; 30; 30
Pure date	0.02	—
Pure jujube	0.03	—
Essential oil	3	—
Cinnamon	0.56	—
BHT	0.06	—
Vit C	0.09	—

D: date; J: jujube; EO: essential oil.

**Table 5:** Average tasting test results.

Visual criteria						Olfactory criteria		Taste criteria					
Formula	Color	Limpidity	Texture	Viscosity	Gloss	Odor intensity	Smell agreeableness	Aroma agreeability	Sugar/acid equilibrium	Bitterness	Astringency	Sugar intensity	Aftertaste
F1	3.65	3.65	4.82	4.04	2.90	4.82	4.14	3.97	3.96	4.22	4.08	4.06	4.08
F2	5.50	4.12	4.37	4.66	4.15	4.37	4.39	4.39	4.32	4.37	4.37	4.36	4.36
F3	3.40	4.37	6.12	4.63	3.45	6.12	5.08	4.82	4.87	5.22	5.00	4.98	5.01
F4	6.47	4.77	4.02	5.09	2.95	4.02	8.52	9.64	10.78	8.24	9.30	9.49	9.45
F5	4.57	4.57	5.67	4.94	4.30	5.67	5.14	5.01	5.03	5.21	5.10	5.09	5.11
F6	6.67	5.37	4.97	5.67	5.05	4.97	5.16	5.21	5.10	5.11	5.15	5.14	5.12
F7	4.50	5.37	6.60	5.49	4.57	6.60	5.81	5.62	5.65	5.92	5.75	5.73	5.76
F8	7.60	5.97	5.62	6.40	5.90	5.62	5.88	5.95	5.84	5.82	5.87	5.87	5.85

## Data Availability

The data used to support the findings of this study are available from the corresponding author upon request.
